# Impact of Newly Diagnosed Left Bundle Branch Block on Long-Term Outcomes in Patients with STEMI

**DOI:** 10.3390/jcm13185479

**Published:** 2024-09-15

**Authors:** Larisa Anghel, Cristian Stătescu, Radu Andy Sascău, Bogdan-Sorin Tudurachi, Andreea Tudurachi, Laura-Cătălina Benchea, Cristina Prisacariu, Rodica Radu

**Affiliations:** 1 Internal Medicine Department, “Grigore T. Popa” University of Medicine and Pharmacy, 700503 Iași, Romania; larisa.anghel@umfiasi.ro (L.A.); bogdan-sorin.tudurachi@d.umfiasi.ro (B.-S.T.); benchea.laura-catalina@d.umfiasi.ro (L.-C.B.); cristina.prisacariu@umfiasi.ro (C.P.); rodica.radu@umfiasi.ro (R.R.); 2 Cardiology Department, Cardiovascular Diseases Institute “Prof. Dr. George I. M. Georgescu”, 700503 Iași, Romania; leonteandreea32@gmail.com

**Keywords:** STEMI, new left bundle branch block, heart failure, long-term outcomes, percutaneous coronary intervention, prognostic, prospective study

## Abstract

**Background/Objectives**: This study assessed the long-term prognostic implications of newly developed left bundle branch block (LBBB) in patients with ST-elevation myocardial infarction (STEMI) and a single coronary lesion, following primary percutaneous coronary intervention (PCI). **Methods:** Among 3526 patients admitted with acute myocardial infarction between January 2011 and December 2013, 42 were identified with STEMI, a single coronary lesion, and newly diagnosed LBBB. A control group of 42 randomly selected STEMI patients without LBBB was also included. All participants were prospectively evaluated with a median follow-up duration of 9.4 years. Demographic, clinical, and laboratory data were analyzed to assess the impact of LBBB on long-term outcomes. **Results:** The baseline characteristics were similar between the groups. The STEMI with new LBBB group had significantly higher rates of new myocardial infarction, revascularization, and mortality, highlighting the severe prognostic implications and elevated risk for adverse outcomes compared to STEMI without LBBB. The multivariate Cox regression analysis demonstrated that the presence of LBBB (HR: 2.15, 95% CI: 1.28–3.62, *p* = 0.003), lower LVEF (HR: 1.45, 95% CI: 1.22–1.72, *p* < 0.001), and longer pain-to-admission time (HR: 1.32, 95% CI: 1.09–1.61, *p* = 0.008) were significant independent predictors of adverse outcomes. **Conclusions:** Newly acquired LBBB in STEMI patients is associated with poorer long-term outcomes. Early identification and management of factors such as reduced LVEF and timely hospital admission, specifically in patients with new-onset LBBB, can improve prognosis.

## 1. Introduction

Acute myocardial infarction with ST-segment elevation (STEMI), one of the most prevalent causes of death and morbidity worldwide, is a life-threatening disorder that requires prompt diagnosis and is frequently the early clinical manifestation of cardiovascular disease. In the assessment of individuals with STEMI, the resting 12-lead electrocardiogram (ECG) serves as the primary diagnostic tool. It is recommended that the ECG results be interpreted as promptly as possible at the first medical contact, ideally within 10 min [1]. Beyond the importance of analyzing the ischemic findings, it is necessary to assess the existence of various conduction disturbances that may occur in the setting of acute coronary syndromes. This is because alterations in the QRS duration and pattern are considered to indicate more acute ischemia and a faster progression of myocardial necrosis compared to changes in the ST-segment alone [2,3,4].

The American Heart Association (AHA) and European Society of Cardiology (ESC) acknowledge the challenge of diagnosing STEMI in the presence of left bundle branch block (LBBB) and determining whether the LBBB is recent or pre-existing, especially when there is no prior electrocardiogram available for comparison [1,5]. Recent studies observed that individuals with bundle branch block (BBB) who had acute myocardial infarction (AMI) had a higher prevalence of three-vessel and left main disease. Also, the occurrence of pulmonary edema, cardiogenic shock, and significant adverse cardiac events is greater in these patients [6,7]. However, other studies revealed no significant associations between the presence of new permanent LBBB or RBBB and the severity of coronary artery atherosclerosis, as measured by Gensini Score (GS) [8].

In the realm of cardiology, the onset of a new LBBB in patients with STEMI represents a pivotal diagnostic and prognostic marker. This study delves into the long-term impact of new LBBB on the incidence of heart failure among STEMI patients, a topic that has garnered attention due to its significant implications for patient outcomes and management strategies.

Our objective was to quantify the specific impact of newly developed LBBB on the long-term prognosis of patients with STEMI and a single coronary lesion who underwent primary percutaneous coronary intervention (PCI). We focused on assessing the prognostic significance of LBBB in terms of mortality risk and the development of heart failure, with the goal of aiding clinicians in optimizing treatment strategies and enhancing patient outcomes.

## 2. Materials and Methods

### 2.1. Patient Recruitment

**Figure 1:**
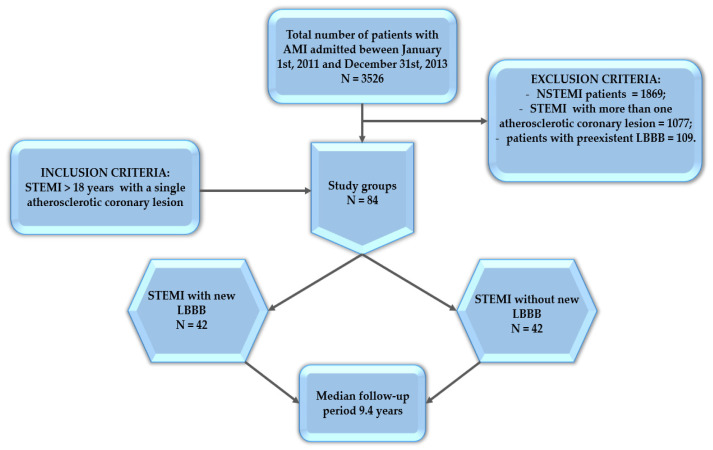
Study flow. AMI, acute myocardial infarction; LBBB, left bundle branch block; NSTEMI, acute myocardial infarction without ST-segment elevation; STEMI, acute myocardial infarction with ST-segment elevation.

This is a prospective observational study, conducted at a primary PCI academic hospital in Eastern Romania, the only hospital in the region with 24 h primary PCI. The study specifically focused on patients aged 18 years or older who met the criteria for STEMI, as defined by the fourth myocardial infarction classification, and a single atherosclerotic coronary lesion [1]. Patients were enrolled consecutively over the study period to minimize selection bias and ensure a representative sample. The inclusion criteria were refined to include only patients with acute STEMI who presented within 12 h of symptom onset. Considering that our hospital is the only primary PCI hospital in the region with 24 h primary PCI services, and given the significant distances between primary PCI centers in this area, we included patients who presented at more than 12 h after symptom onset, but only in the presence of ongoing symptoms suggestive of ischemia, hemodynamic instability, or life-threatening arrhythmias. This inclusion was necessary to reflect real-world clinical practice in our region, where delayed presentation is common due to geographic constraints. STEMI was defined by standard ECG criteria (ST-segment elevation in at least two contiguous leads) and elevated cardiac biomarkers. All participants underwent a prospective evaluation with a median follow-up of 9.4 years. Throughout this period, various biological parameters were assessed to examine the impact of an acute coronary event on lifestyle changes and to evaluate the predictive value of newly developed left bundle branch block (LBBB) regarding mortality and heart failure onset. Patients with type 2 myocardial infarction, defined as myocardial ischemia due to an imbalance between oxygen supply and demand without coronary artery occlusion, as well as those with a prior history of STEMI, PCI, or coronary artery bypass grafting, were excluded from the study (Figure 1).

### 2.2. Data Collection Method

All study participants were interviewed during their hospitalization to document their cardiovascular risk factors. Special attention was placed on accurately recording the precise onset time of chest pain related to acute myocardial infarction. A family history of premature coronary artery disease was defined as the occurrence of sudden death or coronary artery disease in first-degree male relatives under 55 years of age or in first-degree female relatives under 65 years of age.

Peripheral blood samples were collected immediately upon admission of patients to the emergency department or before the urgent coronary angiography, in order to determine the blood cell counts, biochemical analysis of lipids (total cholesterol, low-density lipoprotein cholesterol (LDLc), high-density lipoprotein cholesterol (HDLc), triglycerides), myocardial cytolysis markers (myocardial creatine kinase (CK-MB) and troponin T), glucose metabolism, aspartate aminotransferase (AST), alanine aminotransferase (ALT), and renal function analysis. The glycemic status of the patients was determined using criteria established by the European Diabetes Society: 1. normal glycemic control, defined as fasting blood glucose (FPG) levels below 5.6 mmol/L (100 mg/dL). 2. A diabetes diagnosis was made if patients had two FPG values equal to or greater than 7.0 mmol/L (126 mg/dL) (venous plasma glucose). Patients were classified as diabetic if they had a known history of diabetes, fasting blood glucose levels of 126 mg/dL or higher, or were receiving treatment with oral antidiabetic medications or insulin. These criteria were employed to assess and categorize the glycemic status of the patients in the study [9]. Hypertension was defined as blood pressure measurements equal to or exceeding 140/90 mmHg or a documented history of previous antihypertensive treatment [10]. Body mass index (BMI = weight in kg/height^2^ in m^2^) and smoking status were also evaluated. Obesity was defined as BMI ≥ 30 kg/m^2^.

Electrocardiographic recognition of myocardial infarction in patients with LBBB poses challenges in the emergency department due to the masking effect of ST-segment deviations inherent in LBBB. Various diagnostic criteria have been proposed over the years to aid clinicians in making accurate diagnoses in such cases, with the Sgarbossa criteria being the most widely recognized. The original Sgarbossa criteria require at least 3 points to diagnose STEMI in the presence of LBBB, based on the following: (1) concordant ST-segment elevation of 1 mm or more in at least one lead (5 points); (2) ST-segment depression of 1 mm or more in leads V1-V3 (3 points); and (3) discordant ST-segment elevation of more than 5 mm in at least one lead (2 points). To enhance both sensitivity and specificity, achieving 91% and 90%, respectively, Smith et al. modified the Sgarbossa criteria, and replaced the absolute ST-segment elevation criterion with a relative ST/S ratio of less than 0.25 [3,4]. Patients were classified as having newly developed LBBB if the condition was documented on the initial electrocardiogram at admission, and this condition persisted at the patient’s discharge, without any prior history of LBBB, as confirmed by reviewing previous ECGs when available. Patients with myocardial infarction without prior ECG data were excluded to ensure accurate classification and to include only those with definitively new-onset LBBB. This approach was employed to maintain the accuracy of the classification and the reliability of our findings regarding the impact of new LBBB on long-term outcomes in STEMI patients.

All patients underwent echocardiographic evaluation, with left ventricular ejection fraction (LVEF) measured using the biplane method of discs, based on the modified Simpson’s rule [11].

All patients included in the study underwent cardiac catheterization, and coronary artery stenosis was considered to be present when there was a reduction in the lumen diameter of any of the three coronary arteries or their main branches by more than 50%. The culprit lesion was identified based on angiographic findings consistent with acute thrombus or plaque rupture in a single coronary artery. Coronary arteries with smooth contours and without focal diameter reduction, or those with atherosclerotic lesions causing less than 50% stenosis, were classified as “normal”. Importantly, patients with normal coronaries or non-obstructive disease were excluded from the analysis. Patients with more than one coronary lesion were likely excluded from the study, based on the standard definition of multi-vessel disease, which is defined as stenosis of ≥50% in more than one major coronary artery on coronary angiography. The rationale behind this exclusion was to focus the analysis on single-vessel disease to maintain homogeneity and to reduce confounding variables related to multi-vessel involvement. Also, by excluding patients with multiple lesions, the study aimed to reduce variability in clinical presentation and treatment responses, thus allowing for clearer analysis and comparison between patients with and without new-onset left bundle branch block. These patients were then treated according to established protocols, which involved the placement of second-generation drug-eluting stents. The PCI was customized to suit the coronary anatomy and clinical condition of each individual patient. Following the PCI, all patients received standard treatment regimens that adhered to established guidelines. This included contemporary antiplatelet therapy and standard-dose statin therapy. After discharge, patients were routinely monitored and followed up at the clinic. This comprehensive approach aimed to provide optimal care and management for patients with STEMI.

### 2.3. Follow-Up and Outcomes

During the study, all participants were prospectively evaluated over a median follow-up period lasting 9.4 years. Follow-up assessments were conducted through in-person visits to the clinic. All patients were followed until the study’s completion. The primary endpoint of the study was the incidence of major adverse cardiac and cerebrovascular events (MACEs), defined as a composite of the following outcomes: cardiac mortality (death attributed to cardiac causes), recurrent myocardial infarction (new infarction occurring in the previously treated target vessel), stroke (including both fatal and non-fatal ischemic strokes), and target vessel revascularization (any repeat percutaneous or surgical intervention on the previously treated vessel) [1,2,3,9]. The secondary endpoint of the study aimed to assess the predictive significance of the newly acquired LBBB in terms of both mortality and the development of heart failure.

The study adhered to the principles of the Declaration of Helsinki and its subsequent revisions, as approved by the hospital’s Ethics Committee. Patient data were anonymized, and informed consent for the use of personal information was obtained at the time of hospital admission, ensuring compliance with ethical standards and safeguarding patient confidentiality throughout the research.

### 2.4. Statistical Analysis

First, the normality of the distribution of numerical variables was assessed using the Kolmogorov–Smirnov test. Parametric variables were analyzed with an independent sample t-test, while non-parametric variables were evaluated using the Mann–Whitney U test. Categorical data were compared using the chi-square or Fisher’s exact test as appropriate. A multivariate Cox proportional hazards regression analysis was conducted to identify independent predictors of MACEs. The analysis incorporated key variables, including the presence of LBBB, left ventricular ejection fraction, pain-to-admission time, age, and relevant comorbidities such as diabetes, hypertension, and chronic kidney disease. Initially, each variable was independently analyzed to determine its association with MACE. Variables with a *p*-value of less than 0.05 in the univariate analysis were included in the multivariate Cox regression model. The final Cox regression model was employed to identify independent predictors while accounting for potential confounding factors. The results are presented as hazard ratios (HRs) with 95% confidence intervals (CIs). All statistical analyses were performed using the IBM SPSS statistics version 26.

## 3. Results

Among the 3526 patients admitted with acute myocardial infarction between 1 January 2011 and 31 December 2013, 42 had STEMI with a single coronary lesion and newly diagnosed LBBB. To ensure comparability, a control group of 42 patients with STEMI but without LBBB was randomly selected.

All participants underwent prospective evaluation over a median follow-up period of 9.4 years. During this follow-up, different biological parameters were assessed to investigate the impact of an acute coronary event on lifestyle modifications. Additionally, the study aimed to determine the prognostic significance of newly acquired LBBB in terms of mortality and the development of heart failure.

### 3.1. Patient Characteristics at Baseline

The demographic characteristics, medical history, and admission hemodynamics of the patients were analyzed to determine if there were any significant differences between the groups. The median age of patients with STEMI and new LBBB was 67 years (range 52–83), while for those without LBBB, it was 66 years (range 40–81) with a *p*-value of 0.864, indicating no significant difference. The percentage of female patients was 38.9% in the LBBB group and 33.3% in the control group (*p* = 0.760). This balance in gender distribution helps in isolating the effect of LBBB on outcomes, as gender differences in myocardial infarction can influence prognosis.

Regarding the medical history and admission hemodynamics, there were no significant differences, indicating that the groups were comparable at baseline. The median pain-to-admission time for patients was 9 h (interquartile range [IQR]: 3–13 h) in STEMI with new LBBB and 10 h (IQR: 2–15 h) in patients with STEMI without LBBB. This similarity suggests that both patient groups experienced similar delays from the onset of symptoms to hospital admission. Also, there was a consistency in the location of myocardial infarction between the groups, and more than half of the patients had anterior myocardial infarction. In our study, all procedures were performed via the femoral approach. The interventions were carried out by a team of highly trained and experienced interventional cardiologists, ensuring standardized and expert care throughout the study. All patients underwent successful PCI, with drug-eluting stents, and with a procedural success rate of 100%. Only one patient with new LBBB presented a local hematoma as a post-procedural complication, without significant hemoglobin loss. By ensuring that the two patient cohorts are well-matched at baseline, the study provides a robust platform for investigating the specific effects of newly acquired LBBB on the long-term outcomes of STEMI patients.

Table 1 presents the baseline characteristics of the two groups.

### 3.2. Laboratory Characteristics

The results reveal notable differences and trends that provide insights into the long-term outcomes and management of these patient groups (Table 2).

The hemoglobin and hematocrit levels showed a general trend of slight decline in both patient groups over time. These trends could suggest a mild, chronic anemia or hemodilution developing over time, potentially due to long-term medication use or underlying chronic conditions. A significant reduction in white blood cell counts was observed in both groups, reflecting a possible reduction in systemic inflammation over time. Platelet counts also declined, which might reflect effects of antiplatelet therapies commonly prescribed to these patients.

LDL cholesterol levels showed a substantial decrease in both groups, from 116.9 ± 49.5 to 87.79 ± 31.1 mg/dL, *p* = 0.002 in the LBBB group, and from 123.4 ± 49.16 to 104.19 ± 26.3 mg/dL, *p* = 0.031 in the non-LBBB group. This significant reduction highlights the effectiveness of lipid-lowering therapies and dietary modifications in managing cardiovascular risk factors over the long term, with a better control in patients with LBBB.

Both creatinine and uric acid levels decreased over the follow-up period, suggesting improved renal function or better control of metabolic factors through medication and lifestyle changes. This also might reflect better management of conditions like hypertension and gout, often associated with elevated uric acid.

Glycemic control also showed improvement in both groups, which also indicates successful long-term management of blood glucose levels, possibly through medication, diet, and lifestyle interventions.

### 3.3. Long-Term Outcomes

The presence of new LBBB in STEMI patients significantly influences both therapeutic strategies and prognostic outcomes (Table 3).

#### 3.3.1. Treatment for Heart Failure

Patients with STEMI and new LBBB experienced significant improvements in heart failure management, particularly with the increased use of angiotensin receptor-neprilysin inhibitor/angiotensin-converting enzyme inhibitors/angiotensin II receptor blockers (ARNI/ACEi/ARB) and mineralocorticoid receptor antagonist (MRAs), reflecting a proactive approach in addressing the more severe cardiac dysfunction typically associated with LBBB. The substantial rise in the use of sodium-glucose cotransporter 2 (SGLT2) inhibitors (with 61.90% use for STEMI with new LBBB, *p* < 0.001, and 38.09% use for STEMI without LBBB, *p* = 0.028) in both groups indicates their growing importance in heart failure therapy, underscoring their benefits in reducing hospitalization rates and improving cardiovascular health.

The use of beta-blockers remained high in both groups but did not change significantly over time. This indicates that beta-blockers are a consistently integral part of heart failure management in STEMI patients regardless of the presence of LBBB.

#### 3.3.2. Left Ventricular Ejection Fraction

The analysis of LVEF categories reveals significant shifts over the follow-up period, highlighting improvements in cardiac function. There was a significant decrease in the proportion of patients with severely reduced LVEF (<40%) in both groups. This improvement is indicative of successful heart failure management and potentially better myocardial recovery or remodeling. A significant increase in the proportion of patients with moderately reduced LVEF was observed in patients with STEMI and new LBBB (42.85% vs. 52.38%, *p* = 0.004). In the STEMI without LBBB group, the proportion of patients with preserved LVEF significantly increased (21.42% vs. 38.09%, *p* = 0.026). In contrast, no significant change was observed in the STEMI with new LBBB group (28.57% vs. 26.19%, *p* = 0.456). This shift suggests a transition of patients from severely to moderately impaired cardiac function, reflecting therapeutic efficacy and a better overall recovery in ventricular function among patients without LBBB.

Changes in New York Heart Association (NYHA) functional classification revealed an increase in moderate symptoms in STEMI with new LBBB (NYHA II, 30.95% vs. 42.85%, *p* = 0.003) and consistently low severe symptoms (NYHA IV) in both groups over time.

#### 3.3.3. Outcomes

Long-term outcomes highlight significant differences between the two patient groups, reflecting varying degrees of clinical risk and prognosis (Table 4).

The STEMI with new LBBB group experienced a significant increase in myocardial infarction (*p* < 0.001) and revascularization (*p* < 0.001), compared to the STEMI without LBBB group, indicating a higher risk for recurrent ischemic events and a greater need for interventional strategies. Additionally, there was a trend towards increased stroke incidence (*p* = 0.059) and significantly higher mortality (*p* < 0.001) in the STEMI with new LBBB group, underscoring the severe prognostic implications of new LBBB. In contrast, the STEMI without LBBB group showed no significant changes in these outcomes, suggesting a comparatively lower risk profile.

### 3.4. Predictors of Major Cardiovascular Event in Patients with STEMI and New LBBB

In patients with STEMI, it is very important to identify factors that may influence long-term outcomes.

A multivariate Cox regression analysis was performed to identify independent predictors of major cardiovascular events among STEMI patients with and without LBBB. Variables included in the model were LBBB presence, LVEF, pain-to-admission time, age, and key comorbidities such as diabetes, hypertension, and chronic kidney disease. Each variable was first evaluated independently to assess its association with major adverse cardiovascular events. Significant predictors (*p* < 0.05) were then included in the multivariate analysis. A Cox regression model was applied to identify independent predictors while adjusting for potential confounders. The analysis demonstrated that LBBB (HR: 2.15, 95% CI: 1.28–3.62, *p* = 0.003), lower LVEF (HR: 1.45, 95% CI: 1.22–1.72, *p* < 0.001), and longer pain-to-admission time (HR: 1.32, 95% CI: 1.09–1.61, *p* = 0.008) were significant independent predictors of adverse outcomes (Table 5).

## 4. Discussion

The findings from this study underscore the critical differences in long-term management and outcomes between STEMI patients with new LBBB and those without LBBB. Patients with new LBBB demonstrated significant improvements in heart failure management, particularly with increased use of ARNI/ACEi/ARB and MRAs. However, they also faced higher risks of adverse outcomes, including myocardial infarction, revascularization, and mortality. These results suggest that while therapeutic advancements have improved heart failure management in these patients, the presence of new LBBB remains a marker of poor prognosis, necessitating vigilant monitoring and possibly more aggressive treatment strategies. Conversely, STEMI patients without LBBB showed better overall recovery in ventricular function and lower incidences of adverse outcomes, highlighting a comparatively more favorable long-term prognosis. In the multivariate analysis, the presence of LBBB was independently associated with a higher risk of major adverse cardiovascular events. Moreover, reduced left ventricular ejection fraction and longer pain-to-admission time were also identified as significant independent predictors of poor outcomes.

The baseline characteristics of the two groups were well-balanced, ensuring the reliability of our comparative analyses. There were no significant differences in age, gender distribution, medical history, or admission hemodynamics (*p* > 0.05 for all), which strengthens the reliability of our findings. The balanced distribution of demographic characteristics, comorbid conditions, and initial clinical presentation factors between the groups enhances the reliability of the study’s findings regarding the prognostic impact of LBBB in STEMI patients. This thorough baseline matching allows for a clearer interpretation of the impact of LBBB on long-term outcomes, without being confounded by pre-existing differences between the groups. This careful methodological approach strengthens the validity of the study’s conclusions and supports the clinical relevance of its findings. A relatively small number of patients present with newly developed LBBB in the context of an acute myocardial infarction. For example, in our study, only 1.2% of patients admitted with acute myocardial infarction had STEMI with a single coronary lesion and newly diagnosed LBBB. In a recent study conducted by the Minneapolis Heart Institute STEMI protocol, 3.3% of the patients had either new or apparently new LBBB. Patients with new LBBB were typically older, predominantly female, had lower ejection fractions, and experienced higher rates of cardiac arrest or heart failure compared to those without newly developed LBBB. Additionally, those with new LBBB had a lower incidence of identifiable culprit arteries (54.2% vs. 86.4%, *p* < 0.001). However, they showed a higher rate of all-cause mortality during the one-year follow-up [12]. We acknowledge that our findings offer valuable insights into the specific subgroup of STEMI patients with newly developed LBBB; however, further validation in larger, multicenter studies is required to confirm the broader applicability of these conclusions to the general STEMI population. Despite the limitation of a smaller sample size, our careful selection process and the well-balanced baseline characteristics between the groups help mitigate some concerns related to sample size, thereby enhancing the internal validity of our results.

Our longitudinal follow-up revealed important trends in laboratory parameters. Both groups exhibited a general decline in white blood cell counts, hemoglobin, hematocrit, and platelet counts, suggesting the potential development of mild chronic anemia, reduction in systemic inflammation, and the effects of long-term antiplatelet therapy. Significant reductions in LDL cholesterol levels (*p* = 0.002 for LBBB group, *p* = 0.031 for non-LBBB group) indicate the effectiveness of lipid-lowering therapies in managing cardiovascular risk over time. Effective lipid management is essential for slowing the progression of coronary artery disease and potentially reducing the need for additional reperfusion procedures, thus maintaining vascular health after STEMI [13,14,15]. A recent retrospective study evaluated lipid management in post-MI patients based on the 2019 European Society of Cardiology Guidelines for dyslipidemia. The study found that only 14.7% of patients reached the guideline-recommended LDL-C target of <1.4 mmol/L and achieved a ≥50% reduction from baseline LDL-C at follow-up. This finding underscores the critical need for improved secondary prevention strategies that align with current guidelines [16]. Additionally, improvements in creatinine and uric acid levels point to enhanced renal function and metabolic control, likely due to optimized management of associated conditions. The significant improvements in lipid profiles, glycemic control, and reductions in inflammatory markers underscore the effectiveness of contemporary treatment strategies, including pharmacotherapy and lifestyle modifications.

Significant improvements were observed in heart failure management among patients with STEMI and new LBBB. The increased use of angiotensin receptor-neprilysin inhibitors, ARBs, ACE inhibitors, MRAs, and SGLT2 inhibitors in this group reflects an advanced therapeutic approach aimed at mitigating the more severe cardiac dysfunction associated with LBBB. The substantial rise in the use of SGLT2 inhibitors, in particular, underscores their growing importance in reducing hospitalization rates and enhancing cardiovascular health. LVEF analysis revealed significant improvements in cardiac function over the follow-up period. There was a significant decrease in the number of patients with severely reduced LVEF, with many improving to moderately impaired or preserved LVEF, especially in those without LBBB. This indicates successful heart failure management and better myocardial recovery in these patients. However, patients with newly diagnosed LBBB demonstrated less improvement in left ventricular ejection fraction, underscoring the importance of strict adherence to treatment protocols and regular follow-up to maintain and enhance the health benefits in STEMI patients [17]. Regarding the role of SGLT2 inhibitors in acute coronary syndrome, recent findings provide mixed insights. While preclinical studies suggest these inhibitors may reduce myocardial infarct size and improve cardiac function, clinical data have yet to establish a clear benefit in the ACS setting. Although some benefits, such as reduced contrast-induced acute kidney injury, have been observed, current evidence does not fully support the use of SGLT2 inhibitors in ACS management, regardless of diabetes status [18]. Larger, well-designed RCTs are necessary to clarify their role in this context and potentially expand the therapeutic indications of these drugs. Furthermore, updated clinical guidelines and new evidence have shaped practice patterns, advocating for more personalized treatment strategies and the incorporation of alternative therapies [1,17,18]. For instance, angiotensin receptor-neprilysin inhibitors have demonstrated significant benefits in patients with heart failure, prompting their increased use in contemporary treatment protocols [19,20].

In our study, all procedures were performed via the femoral approach by a highly experienced interventional cardiology team, resulting in a 100% procedural success rate using drug-eluting stents, with only one minor complication (local hematoma) in a patient with new LBBB. A study by Dudek et al. provides valuable insights into the clinical outcomes, over a 12-month follow-up period, following bioresorbable vascular scaffold (BVS) implantation in complex coronary lesions, particularly in the context of acute coronary syndrome. The study showed no significant differences in major adverse cardiovascular events between patients with different levels of vessel tortuosity or calcification. However, patients with ACS, particularly those with unstable angina, experienced a higher rate of target lesion revascularization and device-oriented composite endpoints, suggesting that while BVS can be used effectively in ACS cases, careful patient selection and follow-up are crucial [21].

Our multivariate analysis highlights that the presence of LBBB in STEMI patients contributes to worse long-term outcomes, likely due to the resulting electrical and mechanical dyssynchrony that leads to adverse cardiac remodeling. Reduced left ventricular ejection fraction and delayed pain-to-admission time were also identified as significant independent predictors of poor outcomes. Interestingly, comorbidities such as diabetes, hypertension, and chronic kidney disease were not significant predictors in the multivariate analysis after adjusting for other factors, suggesting that the presence of LBBB, LVEF, and admission delay may have a stronger influence on long-term prognosis in this patient cohort. These results highlight the need for vigilant monitoring and aggressive management strategies in STEMI patients with new LBBB to improve long-term outcomes. Further research could help clarify these relationships and potentially guide more effective treatment strategies. In a large retrospective study of 1875 patients undergoing primary PCI, those with LBBB (n = 155, 8.3%) were significantly older, more often female, and had higher rates of prior MI and CABG compared to patients with STEMI. The LBBB group showed significantly lower rates of acute occlusion (12.2% vs. 63%; *p* < 0.0001) and PCI (26% vs. 83%; *p* < 0.0001). Although 30-day mortality was similar between the groups, overall mortality was significantly higher in the LBBB group during the 2-year follow-up (27.8% vs. 13.9%; *p* = 0.023). These findings highlight the need for improved risk stratification and management strategies for LBBB patients referred for primary PCI [22]. Lahti et al. performed research which found that LBBB was linked to higher cardiac mortality even after accounting for clinical risk variables. However, unlike individuals with non-ischemic ventricular conduction delay (NIVCD), this connection disappeared when LVEF was included in the analysis. These findings provide support for the idea that lower left ventricular ejection fraction (LVEF) caused by mechanical dyssynchrony is a significant unfavorable prognostic factor in LBBB [23].

Nevertheless, a worse prognosis after an acute coronary syndrome (ACS) episode may not only be attributed to the mechanical pumping ability of the heart. NIVCD continued to be a significant predictor of cardiac death even after accounting for in-hospital LVEF. Previously, NIVCD has been linked to a high occurrence of cardiac arrests in STEMI patients undergoing treatment [23,24]. Lee et al. found that people with LBBB and non-ischemic cardiomyopathy (NICD) were more likely to end up in the hospital because of HF, cardiovascular mortality, or death from any cause. Among these patients, those with LBBB had the greatest risk of major adverse cardiovascular events [25]. Nevertheless, the frequency of coronary angioplasty was notably lower in patients with BBB compared to those with normal QRS, which is a key contributing factor to the worse outcomes seen in these groups, especially in patients with LBBB [26]. During a 10-year follow-up of patients with ACS, the presence of LBBB, electrocardiographic left ventricular hypertrophy, and Q waves was shown to be linked with a poorer outcome compared to a normal electrocardiogram, RBBB, ST-segment elevation, or ST-segment depression/T-wave inversion. LBBB was correlated with the most elevated death rates [27]. Patients diagnosed with BBB had more unfavorable baseline features, particularly those with LBBB. During the long time of observation, they also had a more unfavorable result, characterized by an elevated rate of death from all causes [24,25,28].

While our study did not directly utilize cardiac MRI due to the lack of availability in our clinic, we recognize the potential of advanced imaging techniques, such as cardiac MRI, in enhancing risk stratification, particularly for high-risk patients like those with new LBBB. MRI parameters like LVEF and late gadolinium enhancement could complement our identified prognostic factors, providing a more comprehensive evaluation of patient prognosis. This highlights the need for future studies to integrate clinical and imaging data and emphasizes the importance of expanding access to advanced imaging in clinical practice. The findings from our study align with and further emphasize the importance of thorough risk stratification in this patient population. In the DERIVATE-ICM registry, a large multicenter study involving 861 patients with ischemic cardiomyopathy and chronic heart failure, the additional use of cardiac magnetic resonance (CMR) was shown to significantly improve risk stratification for major adverse arrhythmic cardiac events compared to standard transthoracic echocardiography (TTE). The study revealed that key CMR parameters, such as left ventricular end-diastolic volume index, CMR-derived LVEF, and late gadolinium enhancement (LGE) mass, were independent predictors of MAACE. Notably, a multiparametric CMR score provided superior predictive accuracy over the conventional TTE-LVEF cutoff of 35%, resulting in a net reclassification improvement (NRI) of 31.7% (*p* = 0.007). These findings underscore the potential role of advanced imaging techniques, such as CMR, in enhancing the precision of risk assessment and guiding appropriate therapeutic strategies, especially in high-risk patients like those with LBBB who are predisposed to adverse cardiovascular events [29].

The results of another recent study indicate that elevated T2 values in the noninfarcted myocardium (NIM) following STEMI are significantly associated with adverse outcomes. Specifically, patients with higher NIM T2 values (>45 ms) exhibited larger infarct sizes, more microvascular obstruction, and greater left ventricular dysfunction compared to those with lower T2 values. During a median follow-up of 17 months, patients with higher NIM T2 values had a markedly increased risk of MACE, predominantly driven by a significantly higher incidence of myocardial reinfarction (26.3% vs. 1.4%, *p* < 0.001). Multivariable analysis confirmed that elevated NIM T2 values independently predicted MACE (HR: 2.824 [95% CI: 1.254–6.361]; *p* = 0.012), highlighting the prognostic value of this imaging marker. These findings suggest that tissue-level inflammation and edema, as captured by higher NIM T2 values, play a critical role in the post-STEMI pathophysiology and could serve as an important risk stratification tool in this patient population [30].

These studies underscore the critical role of advanced imaging techniques, such as cardiac magnetic resonance, in improving risk stratification for cardiovascular events in high-risk populations. These findings highlight the prognostic value of tissue-level assessments in guiding personalized therapeutic strategies and improving long-term outcomes.

This study has some limitations. The results are based on a relatively small sample size from a single tertiary academic hospital in Romania, which may limit the generalizability of the findings to other populations or healthcare settings. Thus, considering the limited number of patients involved, as a result of the very careful selection process to ensure comparability of results, further validation, including an increase in the number of participants, is necessary to strengthen the findings. The reliance on historical patient records from 2011 to 2013, although the study evaluated the patients for a median follow-up period of almost 10 years, may not fully capture recent therapeutic advances or current management practices. Due to limitations in sample size and the complexity of the dataset, advanced multivariate analyses such as Kaplan–Meier curve generation were not performed. This limitation may impact the generalizability of the findings, and future studies with larger cohorts should consider these analyses to further refine the prognostic significance of LBBB in STEMI patients.

Despite its limitations, this study has several strengths that enhance its credibility and contribute meaningfully to the field of cardiovascular disease management, particularly for patients with complex conditions such as STEMI and newly diagnosed LBBB. The prospective data collection likely improves the accuracy and reliability of the findings compared to retrospective studies. The nearly 10-year follow-up offers valuable long-term insights into the management and outcomes of STEMI patients, both with and without LBBB, which is essential for understanding the chronic nature of coronary artery disease. By focusing on patients with newly developed LBBB, the study addresses a subgroup that is often underrepresented in cardiovascular research but may have poor long-term outcomes, providing specific insights that could shape future guidelines and therapeutic strategies.

## 5. Conclusions

The study highlights the critical prognostic significance of newly acquired LBBB in patients with STEMI. Thus, new-onset LBBB, reduced LVEF, and delayed pain-to-admission time are significant independent predictors of worse long-term outcomes in STEMI patients, highlighting the need for early detection and aggressive management of these factors. Future research should focus on refining therapeutic strategies to improve the prognosis of STEMI patients with LBBB, potentially incorporating advanced heart failure treatments and timely revascularization to mitigate the higher risks observed in this group.

## Figures and Tables

**Table 1:** Patient characteristics at baseline.

Variable	STEMI with New LBBB (n = 42 Patients)	STEMI without LBBB (n = 42 Patients)	*p*-Value
**Demographic characteristics**			
**Age, median (IQR), years**	67 (52–83)	66 (40–81)	0.864
**Female**	38.09%	33.33%	0.760
**Rural area**	40.47%	35.71%	0.630
**Medical history**			
**Hypertension**	69.04%	66.66%	0.822
**Diabetes**	28.57%	23.80%	0.869
**Dyslipidemia**	71.42%	66.66%	0.814
**Heart failure**	23.80%	16.66%	0.685
**Atrial fibrillation**	2.38%	4.76%	0.820
**Chronic kidney disease**	7.14%	9.52%	0.856
**Pain-to-admission time (hours)**			
**<6 h**	16.66%	14.28%	0.565
**6–12 h**	71.42%	76.19%	0.905
**>12 h**	11.90%	9.52%	0.824
**Admission hemodynamics**			
**Killip > I**	23.80%	19.04%	0.855
**Anterior location**	54.76%	57.14%	0.780
**LVEF (%)**	43%	45%	0.856
**Ventricular arrhythmias**	2.38%	4.76%	0.895
**In-hospital outcomes**			
**Median hospital stay (days)**	9 (6–11)	6 (5–9)	**0.048**
**Atrial fibrillation**	7.14%	4.76%	0.780
**Third-degree atrioventricular block**	2.38%	0%	0.855

LBBB, left bundle branch block; LVEF, left ventricular ejection fraction; STEMI, acute myocardial infarction with ST-segment elevation.

**Table 2:** Laboratory characteristics.

Variable	STEMI with New LBBB (n = 42 Patients) Mean ± S.D.		*p*-Value	STEMI without LBBB (n = 42 Patients) Mean ± S.D.		*p*-Value
	Baseline	Follow-Up (Median 9.6 Years)		Baseline	Follow-Up (Median 9.2 Years)	
**Hemoglobin (g/dL)**	13.80 ± 1.37	13.44 ± 1.44	0.247	14.58 ± 1.28	14.20± 1.40	0.201
**Hematocrit (%)**	43.65 ± 5.6	42.55 ± 6.7	0.419	46.55 ± 4.8	44.50 ± 6.6	0.111
**Platelets (mm^3^)**	285.200 ± 11,500	275,000 ± 10,340	**<0.001**	245,000 ± 10,680	234,050 ± 10,450	**<0.001**
**White blood cells (mm^3^)**	14,500 ± 1065	10.230 ± 1050	**<0.001**	12,350 ± 1055	10,050 ± 1060	**<0.001**
**LDL cholesterol (mg/dL)**	116.9 ± 49.5	87.79 ± 31.1	**0.002**	123.4 ± 49.16	104.19 ± 26.3	**0.031**
**Creatinine (mg/dL)**	1.1 ± 0.4	0.9 ± 0.6	0.080	1.2 ± 0.8	1.0 ± 0.5	0.177
**Uric acid (mg/dL)**	6.9 ± 0.9	6.2 ± 0.8	**0.001**	7.1 ± 0.8	6.7 ± 0.8	**0.027**
**Glycemia (mg/dL)**	123 ± 43	106 ± 23	**0.029**	107 ± 11	102 ± 31	0.330

LDL, low-density lipoprotein; LBBB, left bundle branch block; STEMI, acute myocardial infarction with ST-segment elevation.

**Table 3:** Long-term treatment and functional outcomes in patients with STEMI, with and without LBBB.

Variable	STEMI with New LBBB (n = 42 Patients)		*p*-Value	STEMI without LBBB (n = 42 Patients)		*p*-Value
	Baseline	Follow-Up (Median 9.6 Years)		Baseline	Follow-Up (Median 9.2 Years)	
**Treatment for heart failure**						
**Beta-blockers**	92.85%	85.71%	**0.467**	78.57%	85.71%	0.238
**ARNI/ACEi/ARB**	0/33.3/28.57%	42.85/7.14/4.76%	**0.040**	0/57.14/21.42%	38.09/4.76/19.04%	0.560
**MRA**	14.28%	21.42%	**0.031**	11.90%	14.28%	0.670
**SGLT2i**	0%	61.90%	**<0.001**	0%	38.09%	**0.028**
**Diuretics**	59.52%	40.47%	0.208	14.28%	11.90%	0.450
**Left ventricular ejection fraction (%)**						
**<40%**	30.95%	21.42%	**<0.001**	14.28%	7.14%	**<0.001**
**40–50%**	42.85%	52.38%	**0.004**	64.28%	54.76%	**0.003**
**>50%**	28.57%	26.19%	0.456	21.42%	38.09%	**0.026**
**Heart failure**						
**NYHA I**	2.38%	4.76%	0.445	4.76%	7.14%	0.767
**NYHA II**	30.95%	42.85%	**0.003**	23.80%	28.57%	0.522
**NYHA III**	7.14%	4.76%	0.767	4.76%	2.38%	0.445
**NYHA IV**	2.38%	0%	0.497	2.38%	0%	0.497

ARNI/ACEi/ARBs, angiotensin receptor-neprilysin inhibitor/angiotensin-converting enzyme inhibitors/angiotensin II receptor blockers; LBBB, left bundle branch block; MRA, mineralocorticoid receptor antagonist; NYHA, New York Heart Association; SGLT2i, sodium-glucose cotransporter 2 inhibitors; STEMI, acute myocardial infarction with ST-segment elevation.

**Table 4:** Comparison of long-term outcomes in patients with STEMI with and without LBBB, at baseline and follow-up.

Variable	STEMI with New LBBB (n = 42 Patients)		*p*-Value	STEMI without LBBB (n = 42 Patients)		*p*-Value
	Baseline	Follow-Up (Median 9.6 Years)		Baseline	Follow-Up (Median 9.2 Years)	
Outcomes						
**Myocardial infarction**	0	9.52%	**<0.001**	0	4.76%	0.059
**Revascularization**	0	9.52%	**<0.001**	0	4.76%	0.059
**Stroke**	0	4.76%	0.059	0	2.38%	0.497
**Death**	0	9.52%	**<0.001**	0	4.76%	0.059

LBBB, left bundle branch block; STEMI, acute myocardial infarction with ST-segment elevation.

**Table 5:** Univariate and multivariate Cox regression analysis to estimate predictors of MACE in STEMI patients with and without LBBB.

Variable	Univariate			Multivariate		
	HR	95% CI	*p*	HR	95% CI	*p*
**LBBB presence**	2.6	1.45–4.68	0.002	2.15	1.28–3.62	**0.003**
**LVEF (%)**	1.7	1.30–2.21	<0.001	1.45	1.22–1.72	**<0.001**
**Pain-to-admission time (hours)**	1.4	1.15–1.71	0.004	1.32	1.09–1.61	**0.008**
**Age (years)**	1.1	0.98–1.25	0.076	1.05	0.94–1.18	0.342
**Diabetes**	1.6	1.05–2.45	0.031	1.35	0.90–2.02	0.120
**Hypertension**	1.2	0.78–1.85	0.384	-	-	-
**Chronic kidney disease**	1.85	1.11–3.08	0.018	1.48	0.90–2.45	0.125

CI, confidence interval; HR, hazard ratio; LBBB, left bundle branch block; LVEF, left ventricular ejection fraction.

## Data Availability

All data generated or analyzed during this study are included in this article.
